# Enhancing Liver Delivery of Gold Nanoclusters via Human Serum Albumin Encapsulation for Autoimmune Hepatitis Alleviation

**DOI:** 10.3390/pharmaceutics16010110

**Published:** 2024-01-14

**Authors:** Cong Meng, Yu Liu, Yuping Ming, Cao Lu, Yanggege Li, Yulu Zhang, Dongdong Su, Xueyun Gao, Qing Yuan

**Affiliations:** 1 Center of Excellence for Environmental Safety and Biological Effects, Beijing Key Laboratory for Green Catalysis and Separation, Department of Chemistry, Beijing University of Technology, Beijing 100124, China; mengcong@emails.bjut.edu.cn (C.M.); yupingming@emails.bjut.edu.cn (Y.M.); lucaobjut@163.com (C.L.); liliygg@163.com (Y.L.); lyz092800@emails.bjut.edu.cn (Y.Z.); gaoxy@ihep.ac.cn (X.G.); 2 College of Biological and Chemical Engineering, Qilu Institute of Technology, Jinan 250200, China; liuyu360@qlit.edu.cn

**Keywords:** peptide-conjugated gold nanoclusters, autoimmune hepatitis, liver delivery, human serum albumin encapsulation, IFN-γ/STAT

## Abstract

Peptide-protected gold nanoclusters (AuNCs), possessing exceptional biocompatibility and remarkable physicochemical properties, have demonstrated intrinsic pharmaceutical activity in immunomodulation, making them a highly attractive frontier in the field of nanomedicine exploration. Autoimmune hepatitis (AIH) is a serious autoimmune liver disease caused by the disruption of immune balance, for which effective treatment options are still lacking. In this study, we initially identified glutathione (GSH)-protected AuNCs as a promising nanodrug candidate for AIH alleviating in a Concanavalin A (Con A)-induced mice model. However, to enhance treatment efficiency, liver-targeted delivery needs to be improved. Therefore, human serum albumin (HSA)-encapsulated AuNCs were constructed to achieve enhanced liver targeting and more potent mitigation of Con A-induced elevations in plasma aspartate transaminase (AST), alanine transaminase (ALT), and liver injury in mice. In vivo and in vitro mechanism studies indicated that AuNCs could suppress the secretion of IFN-γ by Con A-stimulated T cells and subsequently inhibit the activation of the JAK2/STAT1 pathway and eventual hepatocyte apoptosis induced by IFN-γ. These actions ultimately protect the liver from immune cell infiltration and damage caused by Con A. These findings suggest that bio-protected AuNCs hold promise as nanodrugs for AIH therapy, with their liver targeting capabilities and therapeutic efficiency being further improved via rational surface ligand engineering.

## 1. Introduction

Autoimmune hepatitis (AIH) is a severe immune-mediated liver disease characterized by the immune system’s attack on liver cells, leading to liver inflammation and fibrosis [1]. The primary clinical features of AIH include interface hepatitis, hypergammaglobulinemia, elevated transaminase levels, hepatocyte necrosis, and the presence of specific autoantibodies [2,3]. AIH has a global prevalence and incidence that have been progressively increasing in recent years [4]. Failure to promptly and effectively manage AIH can result in complications such as cirrhosis and liver failure, posing a significant threat to the lives of patients [5]. Glucocorticoids and immunosuppressants are the primary therapeutic interventions for AIH; however, their conventional utilization and patient compliance are impeded by the adverse effects and drug resistance they entail [1]. Consequently, there exists a pressing necessity to explore novel drug candidates with promising efficacy and minimal toxicity.

Despite an incomplete understanding of the precise pathogenesis and etiology of AIH, it has been demonstrated that the disturbance of T cell and macrophage-mediated autoimmune equilibrium is strongly associated with the onset of AIH [6,7,8]. Notably, the pathogenesis and pathological alterations observed in mice with Concanavalin A (Con A)-induced hepatitis closely resemble those observed in human AIH [8]. Studies have indicated that Con A activates immature CD4^+^ T cells and macrophages in vivo and triggers several signaling pathways to induce immune-mediated liver injury [8]. After Con A stimulation, the infiltration of CD4^+^ T cells and macrophages in liver tissue increased, promoting the secretion of interferon-gamma (IFN)-γ, tumor necrosis factor (TNF)-α, interleukin 6 (IL-6), and other cytokines [9,10,11]. Hepatocyte apoptosis or necrosis induced by these inflammatory cytokines is the main cause of Con A-induced liver damage, and the extensive flaky liver necrosis leads to significant elevations of alanine aminotransferase (ALT) and aspartate aminotransferase (AST) [12]. IFN-γ is a major cytokine that activates the Janus kinase (JAK)/signal transducer and activator of the transcription (STAT) pathway in liver cells to activate programmed cell death [13]. IFN-γ antibody therapy significantly protects the liver from Con A-induced damage, demonstrating that IFN-γ is an important therapeutic target of AIH [8]. TNF-α and IL-6 also play key roles in promoting inflammatory cell infiltration in the liver and inducing hepatocyte apoptosis to mediate liver injury [14,15].

Compared to traditional drugs, nanomedicines have exhibited several advantages, such as a reduction in adverse events, enhanced capabilities for targeted delivery, and improved biosafety [16]. Gold nanoclusters (AuNCs) protected by peptides or proteins have shown great potential for therapeutic applications and have become an attractive frontier in nanomedicine due to their supernormal physicochemical and biomedical properties, such as ultrasmall size, atomically precise composition, strong luminescence, tailorable surface chemistry, water-soluble, excellent biocompatibility and intrinsic pharmaceutical activities [17,18,19,20]. Especially in recent years, the immunoregulatory activity of AuNCs was revealed in tumor immunotherapy and autoimmune disease therapy, including rheumatoid arthritis, psoriasis, and inflammatory bowel disease [21,22,23,24]. The mechanism of action is associated with the inhibition of the activated NF-κB pathway [21,22,23]. NF-κB plays an important role in the pathogenesis of AIH, and the elevation of IFN-γ induced by Con A in T cells is associated with increased NF-κB activation [25,26,27]. The findings suggest that AuNCs may have potential efficacy in attenuating AIH. However, the activity of AuNCs in AIH therapy has not yet been explored, and how to achieve liver targeting to promote therapeutic applications is also a valuable issue.

Surface ligands are crucial for the biomedical applications of bio-protected AuNCs, and surface engineering can trigger significant changes in the physicochemical and biological properties of AuNCs, such as water solubility, stability, biodistribution, pharmacokinetics, and excretion, to achieve greater efficacy [28,29,30]. For instance, the biodistribution and toxicity of bovine serum albumin (BSA)- and glutathione (GSH)-protected AuNCs have been comprehensively compared in murine models [31]. BSA-protected AuNCs showed extremely higher bioaccumulation in the liver than the GSH-protected AuNCs but did not cause obvious impacts on normal liver functions at a very high dose (7.55 mg/kg), suggesting that the surface chemistry of albumin may be used to enhance the targeted therapy of AuNCs for liver diseases [31].

**Scheme 1:**
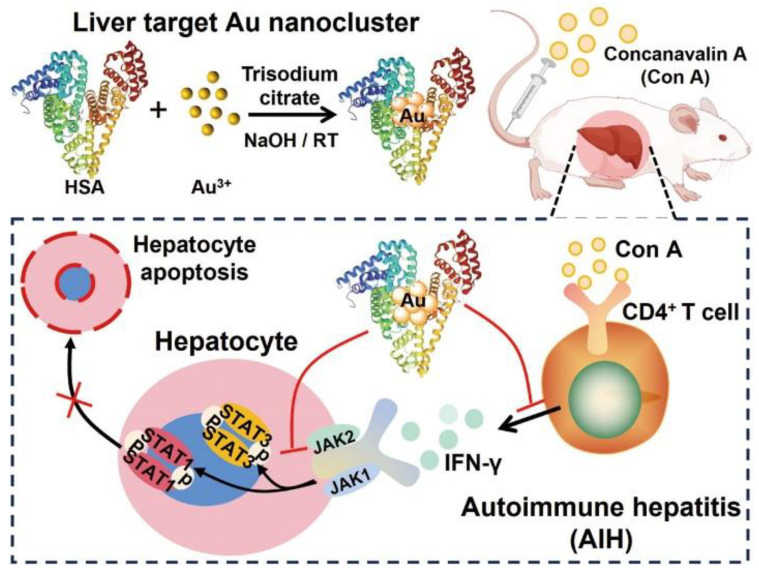
Schematic illustration of HA construction for liver targeting and AIH attenuating.

Human serum albumin (HSA) is the homologous protein of BSA, and its sequence and structure are highly similar to BSA. Moreover, HSA is an FDA-approved drug delivery system (DDS) that has been used in the formulation of paclitaxel (Abraxane), indicating its excellent biosafety and clinical translational promise [32]. In this study, we first explored the potential activity of AuNCs in AIH treatment by using well-defined GSH-protected AuNCs (named GA) and then constructed an HSA-encapsulated AuNCs (named HA) with preferred liver targeting to improve the therapeutic efficacy for AIH. Results indicated that HSA-encapsulated Au_25_ nanoclusters with strong red fluorescence were obtained, which showed obviously higher liver targeting compared to GA and significantly improved the therapeutic efficacy for AIH in Con A-injected mice (Scheme 1). In vivo and in vitro mechanism studies showed that HA could suppress the overexpression and secretion of IFN-γ and IL-6 in Con A-stimulated T cells and then subsequently inhibit the activation of JAK2/STAT1 pathway and eventual cell apoptosis induced by IFN-γ in hepatocytes, thereby protecting the liver from Con A-induced immune cell infiltration and liver damage. These findings indicate that bio-template AuNCs may be a promising nanodrug for AIH, and its liver targeting efficiency and therapeutic activity can be improved via surface ligand engineering.

## 2. Materials and Methods

### 2.1. Materials

Glutathione (GSH, γ-Glu-Cys-Gly) and human serum albumin (HSA) were obtained from Sigma-Aldrich (purity: 95%, St. Louis, MO, USA); hydrogen tetrachloroaurate trihydrate (HAuCl_4_·3H_2_O) was also purchased from Sigma-Aldrich. Dulbecco’s Modified Eagle Medium/Nutrient Mixture F-12 (DMEM/F12) and fetal bovine serum (FBS) were purchased from Gibco (Waltham, MA, USA). The CCK-8 kit (Cell Counting Kit 8) was acquired from Dojindo Laboratories (Kumamoto, Japan). The recombinant Mouse IFN gamma (IFN-γ) protein was obtained from Novoprotein (Suzhou, China). The enzyme-linked immunosorbent assay (ELISA) assay kits for IFN-γ, IL-6, and TNF-α were obtained from Mreda Company (Beijing, China). Antibodies against p-STAT1/STAT1, STAT3, β-actin, and HRP-labeled Goat Anti-Rabbit IgG (H + L) second antibody were purchased from Beyotime Biotechnology (Shanghai, China). Antibodies against p-STAT3, Caspase3, p-JAK2, and JAK2 were purchased from Bioss (Beijing, China).

### 2.2. Synthesis of GSH-Protected AuNCs (GA)

An amount of 100 mL freshly dissolved GSH (30 mM) was mixed with an equal volume of HAuCl_4_·3H_2_O solution (20 mM) under stirring (500 rpm) at room temperature for 10 min. The mixture was heated to 70 °C for 12 h in a water bath with gentle stirring (500 rpm). Then, the reaction solution was kept at room temperature and shielded from light for another 12 h. After that, the generated orange-emitting AuNCs were purified according to our previous report [21]. The last step is to further purify the AuNCs aqueous solution using ultrafiltration (Millipore, Burlington, MA, USA, MWCO: 3 kDa) to remove the unreacted free ions and stock at 4 °C.

### 2.3. Synthesis of HSA-Encapsulated AuNCs (HA)

Under optimized conditions, 2 mL of HSA (10 mgmL^−1^) in ultrapure water was transferred to 2 mL of HAuCl_4_ (4.375 mM Au) under constant stirring. After 2 min, 0.5 mL NaOH solution (0.5 M) was added to the mixed solution, wrapped in aluminum foil to avoid light, and stirred continuously at room temperature for 12 h. After the reaction was complete, the pH of the solution was adjusted to 7 with hydrochloric acid, and then the bacteria and impurities were filtered out with a 0.22 μm filter. The solution obtained from the reaction was subjected to ultrafiltration with a 10 KDa ultrafiltration tube (Millipore, MWCO: 10 kDa), centrifuged for 15 min, and concentrated to deep brown with a volume of about 1 mL. The purified sample was stocked at 4 °C for subsequent experiments.

### 2.4. Characterization of the Au Nanoclusters

The photoluminescence (PL) spectra of as-prepared AuNCs were measured by a fluorescence spectrophotometer (Shimadzu RF-5301, Kyoto, Japan). The absorption spectrum of HSA and HA was detected by a UV−vis spectrometer (UV-2600, Shimadzu, Japan). The hydrate particle sizes of the as-prepared AuNCs were analyzed by dynamic light scattering (Malvern Zetasizer Nano S, Malvern, UK). The dispersion, element composition, and core size of HA were determined by a scanning transmission electron microscope (STEM) combined with energy dispersive spectrum (EDS) (FEI Talos F200X-G2). The content of Au in the obtained HA clusters was quantified by inductively coupled plasma mass spectrometry (ICP-MS, PerkinElmer, Waltham, MA, USA). The molecular weight of the HSA-encapsulated AuNCs was analyzed by matrix-assisted laser desorption/ionization time of flight mass spectrometry (MALDI-TOF-MS) in positive ion linear mode (ABI MALDI-TOF system), with sinnapinic acid as the matrix.

### 2.5. Experiments In Vitro

#### 2.5.1. Culture and Treatment of Cells

Jurkat cells and Alpha mouse liver 12 (AML12) cells were cultured in DMEM/F12 medium supplemented with 10% heat-inactivated FBS and 100 µg mL^−1^ penicillin/streptomycin, in a 37 °C incubator containing 5% CO_2_. The cells in the logarithmic growth phase were used for further evaluation. 

#### 2.5.2. Cell Viability Assay with Cell Counting Kit-8

The cytotoxicity of HA to Jurkat and AML12 cells was determined by CCK-8 assay. The cells were seeded in 96-well plates at a density of 1 × 10^4^ cells per well and cultured for 24 h. Then, different concentrations of HA (5, 20, 50, and 100 µM Au) were added to the wells and incubated for 24 h. The medium was removed, and 100 µL fresh medium containing 10% (*v*/*v*) CCK-8 was added. The cells were incubated with CCK-8 for 1 h at 37 °C. The absorption of OD_450_ was detected by a microplate reader (SpectraMax M4, Molecular Devices, Sunnyvale, CA, USA).

#### 2.5.3. Protein Extraction and Western Blotting

AML12 cells were seeded in a 6-well plate at a density of 2 × 10^5^ cells per well and incubated overnight. Then, different concentrations of HA (5, 20, 50, and 100 µM Au) were added and incubated for 2 h. Subsequently, IFN-γ (50 ng/mL) was introduced to stimulate the cells for 30 min. The whole proteins of the treated AML12 cells were extracted with RIPA Lysis Buffer and quantified by BCA Protein Assay Kit (Beyotime Biotechnology, Shanghai, China). An equal amount of protein in each sample was electrophoretically separated in 10% SDS-PAGE gel and then transferred to PVDF membranes. After blocking with Blocking Buffer (Beyotime Biotechnology, Shanghai, China) for 1 h at room temperature, the membranes were incubated with indicated antibodies at 4 °C overnight. After that, the secondary antibody was incubated with the membranes for 1 h at room temperature. The immunoreactive bands in the membrane were visualized by the enhanced chemiluminescence (ECL) reagent (GE Healthcare London, UK) and imaged by a gel imaging system (Tanon, Shanghai, China).

### 2.6. Experiments In Vivo

#### 2.6.1. Animal Studies

All animal experiments were approved by the Ethics Committee of Beijing University of Technology (approval number: HS202209003) and were conducted in strict accordance with the National Law on the Use of Experimental Animals (China). Female BALB/c mice were acclimated for 3 days and randomly assigned into three or four groups (5 per group): Control; Con A; and GA treatment (add HA treatment group in the comparative experiment). Con A was dissolved in pyrogen-free PBS, and then it was injected intravenously into the Con A and treatment groups at a dose of 12.5 mg/kg body weight once a week to induce hepatitis for 4 weeks. The treatment group received intraperitoneal injections of GA or HA at a dose of 7.5 mg Au/kg on alternate days 14 times (28 days). In contrast, the Control group received equivalent volumes of normal saline injections for comparative purposes. Throughout the experimental period, meticulous attention was paid to monitoring the phenotypic changes in the mice, and their body weights were recorded daily. Following the final Con A injection, 8 h post-administration, the mice were euthanized promptly. Serum and major organs were collected after the mice were sacrificed. The liver and other major organs were weighed, and the organ coefficient was obtained by dividing organ weight by body weight. A portion of the liver tissue was immediately fixed in paraformaldehyde for subsequent Hematoxylin and Eosin (HE) staining experiments, while another segment was flash-frozen at −80 °C for further analysis. This methodology allowed for a comprehensive evaluation of the induced liver injury and its subsequent analysis in the context of the autoimmune hepatitis model.

#### 2.6.2. Quantitative Detection of Au Distribution In Vivo by ICP-MS

At the end of the animal experiment, major organs, including the heart, liver, spleen, lung, and kidney, were harvested. A quarter portion of each organ was lyophilized, weighed, and pre-digested with a mixture of H_2_O_2_ and HNO_3_ (1:3, *v*/*v*) overnight until visible tissue blocks completely disappeared. The sample was further digested using aqua regia (HCl: HNO_3_ = 1:3, *v*/*v*) at 160 °C. The remaining 0.1–0.2 mL sample solution was diluted with 1% HCl and 2% HNO_3_ to a final test volume. The sample solution was analyzed for Au content using ICP-MS (Thermo Elemental X7, Waltham, MA, USA), and high-grade pure bismuth nitrate (Bi concentration = 1 mg/mL) was used as the internal reference. A series of standard solutions containing Au elements at different concentrations (0.1 ppb, 0.5 ppb, 1 ppb, 5 ppb, 10 ppb, 50 ppb, and 100 ppb) were selected to prepare the standard curve.

#### 2.6.3. Enzyme-Linked Immunosorbent Assay

The levels of AST, ALT, IFN-γ, IL-6, and TNF-α in serum were assessed by mouse enzyme-linked immunosorbent assay (ELISA) kits. For example, to exemplify the detection process of AST, a double antibody sandwich method was employed. Initially, purified AST was immobilized onto a solid-phase antibody. Subsequently, samples containing AST were added, followed by the addition of AST antibody labeled with an HRP tag, resulting in the formation of an “antibody–antigen–enzyme antibody” complex. Finally, a TMB substrate was introduced, inducing a color reaction. By analyzing the intensity of the resultant color, a semi-quantitative assessment was made by measuring the absorbance value, allowing for the calculation of AST content in the sample.

#### 2.6.4. Liver Tissue Sections for HE Staining

Mouse liver tissue samples were collected and fixed in a solution containing 4% paraformaldehyde. The fixed liver tissue was subsequently embedded in paraffin, cooled, and solidified into blocks, which were then sectioned into slices measuring 5 μm in thickness. To ensure optimal penetration of the dye solution, the liver tissue sample sections were dewaxed using xylene. Following dewaxing, the liver tissue samples were immersed in a series of ethanol solutions with varying concentrations, each for a duration of 5 min, to achieve proper hydration. Subsequently, staining was performed utilizing hematoxylin and eosin (H&E), and excess dye was eliminated by washing. The tissue sections were then dehydrated using a gradient of ethanol solutions, rendered transparent using xylene, and sealed using neutral gum. Finally, representative regions were carefully selected under a microscope, and a qualitative analysis of inflammatory cell responses was conducted at a magnification of 40×. Additionally, a professional investigator carried out a quantitative analysis of inflammatory infiltration and tissue damage in the liver tissue. The pathological scores of liver injury were assessed by grading each section of the liver from 0 to 4 according to the extent of the lesion, including hepatocyte necrosis, acidophilic degeneration, and inflammatory cell infiltration; a higher score indicated a more severe lesion: score 0 = No obvious abnormality; score 1 = slight abnormality; score 2 = mild abnormality; score 3 = moderate abnormal; score 4 = severe abnormality. Each liver section was graded, and a mean score was given for each group.

#### 2.6.5. Immunofluorescence Experiments

To evaluate the degree of hepatocyte apoptosis, liver sections were stained with a TdT-mediated dUTP Nick-End Labeling (TUNEL) kit according to the manufacturer’s recommendations. The sections were then analyzed under a fluorescence microscope. Green fluorescence indicates apoptotic cells. Paraffin-embedded liver tissue sections underwent a deparaffinization process via three sequential immersions in xylene, each lasting 8 min. Subsequently, they were progressively rehydrated in alcohol solutions with decreasing concentrations of 100%, 90%, 80%, and 60%, each for a duration of 8 min. Following this, the sections were subjected to treatment with a 3% hydrogen peroxide solution for a 15-min period at ambient room temperature, and a subsequent repair step involved exposure to an EDTA solution with a pH of 8.0 for an additional 3 min. To mitigate non-specific binding, the sections were incubated with goat serum for 30 min at 37 °C. Following this blocking step, the sections were subjected to an immunolabeling procedure involving the use of specific primary antibodies (F4/80, p-STAT1, p-STAT3) at a temperature of 4 °C overnight. On the subsequent day, the sections underwent thorough washing in phosphate-buffered saline (PBS) three times, each for a duration of 5 min, and were subsequently incubated with secondary antibodies at 37 °C for 20 min in a well-illuminated environment. After three successive PBS washes conducted in the dark, the sections were counterstained with DAPI at a concentration of 1 μg/mL for a duration of 30 min. The specimens were then sealed with a suitable mounting medium and subjected to photography and observation under a fluorescence microscope.

### 2.7. Statistical Analyses

All experimental results in this paper were expressed as mean ± standard deviation (SD). One-way analysis of variance (one-way ANOVA) was used between the experimental group (intervention group) and the control group, and *p* < 0.05 was considered statistically significant. The software used for all data processing was SPSS version 19.0 (Chicago, IL, USA).

## 3. Results and Discussion

### 3.1. GSH-Protected AuNCs Attenuates Con A-Induced Liver Injury in Mice

**Figure 1:**
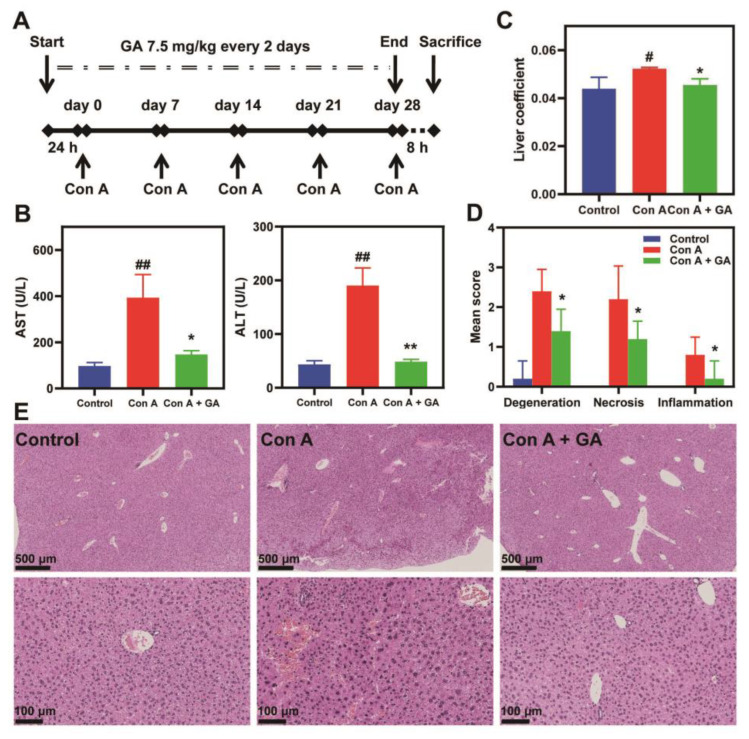
GA attenuates Con A-induced liver injury in mice. (**A**) Procedure of GA treatment in Con A-induced AIH mice. (**B**) ALT and AST levels in serum determined by ELISA assay. (**C**) Liver coefficient of mice in each group. Data are presented as the mean ± SD (*n* = 5). # *p* < 0.05, ## *p* < 0.01 was compared with the Control group. * *p* < 0.05, ** *p* < 0.01 was compared with the Con A injected group. (**D**) Pathology score analysis of Con A-induced hepatocyte necrosis, acidophilic degeneration, and inflammatory cell infiltration. Data are presented as the mean ± SD (*n* = 5). * *p* < 0.05, ** *p* < 0.01 was compared with the Con A-injected group. (**E**) H&E staining of liver sections. The upper row of images shows the overall profile in 50× magnification (Scale bar = 500 μm). The lower row of images displays the representative areas with greater magnification (scale bar = 100 μm).

To assess the potential ameliorating activity of bio-protected gold nanoclusters (AuNCs) against AIH, we first evaluated the therapeutic activity of GSH-protected AuNCs (GA) in Con A-induced mouse hepatitis [8]. The red fluorescent GA was prepared according to our previously reported method and verified by fluorescence spectra and dynamic light dispersion (DLS) characterization (Figure S1) [21,22]. To more closely resemble chronic autoimmune hepatitis, mice received five tail vein injections of Con A (12.5 mg/kg) once a week, and livers and serum were collected 8 h after the last injection of Con A for subsequent analysis [33]. GA (7.5 mg/kg) was pre-treated intraperitoneally the day before the first injection of Con A and then administered every two days (Figure 1A). As shown in Figure 1B, Con A injection induced significant increases in serum ALT and AST, which was effectively eliminated by GA treatment. In addition, the elevation of the liver coefficient caused by multiple injections of Con A was also obviously inhibited by GA (Figure 1C). Histopathological analysis of livers demonstrated that Con A stimulation induced a large number of lymphocytic infiltrates in the portal tract, multiple flaky necrotic areas, and acidophilic degeneration in mouse liver (Figure 1D,E). Compared with the model group, histopathological observation showed that GA treatment remarkably alleviated liver damage caused by Con A (Figure 1E). Pathology score statistics indicated that Con A-induced hepatocyte necrosis, acidophilic degeneration, and inflammatory cell infiltration can be significantly reduced by GA treatment (Figure 1D). These results demonstrated a potential liver protective activity of bio-protected AuNCs in immune-mediated liver injury.

### 3.2. Preparation and Characterization of HSA Encapsulated AuNCs

**Figure 2:**
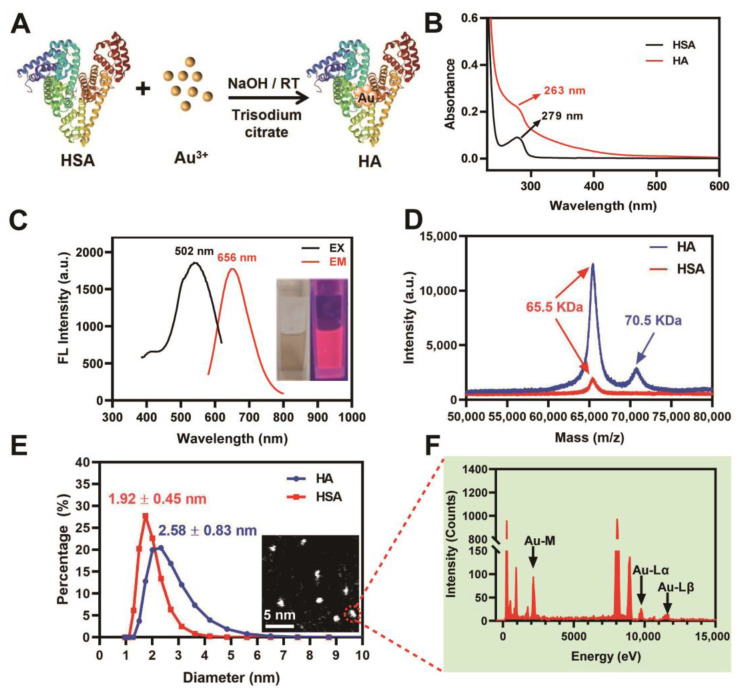
Characterization of HA. (**A**) Diagram of the synthesis of HA. (**B**) Ultraviolet-visible absorption spectrum characterization of HA and HSA. (**C**) Fluorescence spectrum of HA, excitation peak at 502 nm, and emission peak at 656 nm. Inset is the image of HA solution under sunlight (left) and UV light (right), respectively. (**D**) MALDI-TOF-MS analysis of HA in positive ion linear mode. (**E**) Dynamic light scattering (DLS) analysis of HA. The inset represents the scanning transmission electron microscope (STEM) image of HA (Scale bar = 5 nm). (**F**) EDS analysis of Au element from the STEM identified HA particle.

HSA-based nanoparticles can accumulate rapidly in the liver after injection in vivo. We hypothesized that has-encapsulated AuNCs might increase liver accumulation and, thus, exhibit higher AIH therapeutic efficiency than GA. Therefore, an HSA-encapsulated AuNC was prepared and named HA (Figure 2A) [34,35]. The Ultraviolet-visible (UV-VIS) absorption spectrum showed that after the reaction, the absorption peak of HSA at 279 nm shifted to 263 nm (Figure 2B). The obtained HA solution is deep brown under sunlight and emits strong red fluorescence under ultraviolet excitation (Figure 2C inset). Fluorescence spectrum analysis indicated the excitation and emission peaks of HA were located at 502 nm and 656 nm, respectively (Figure 2C). The molecular weights of HSA and HA determined by Matrix-assisted laser desorption/ionization time-of-flight (MALDI-TOF) mass spectrometry were 65.5 KDa and 70.5 KDa, respectively (Figure 2D). The peak shift of about 5 kDa from HSA to HA can be attributed to the presence of 25 Au atoms in AuNCs, which is consistent with the previously reported BSA-AuNCs [34]. The zeta potential of HA was about −50 mV, indicating its good colloidal stability (Figure S2). Dynamic light scattering (DLS) measurement showed that the average hydrodynamic particle size of HA was about 2.58 nm, while the size of HSA was about 1.92 nm (Figure 2E). Scanning transmission electron microscope (STEM) and energy dispersive spectrum (EDS) analysis of Au element further confirmed the successful synthesis of HA (Figure 2E inset and Figure 2F).

### 3.3. HA-Enhanced Liver Targeting and Attenuation in Con A-Induced AIH

**Figure 3:**
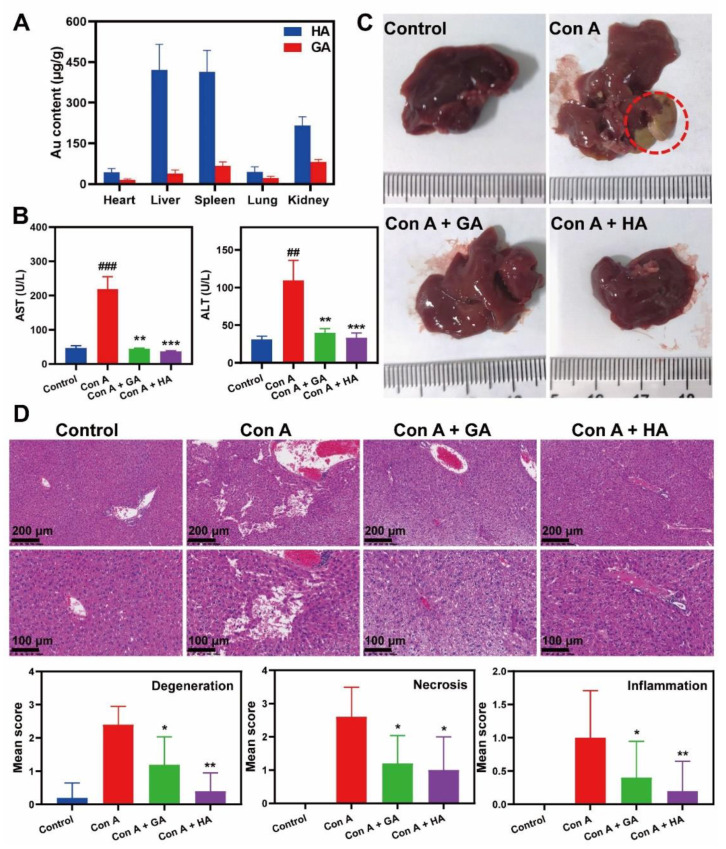
Liver targeting and therapeutic activity of HA. (**A**) The biodistribution of Au quantified by ICP-MS. (**B**) The levels of ALT and AST in serum of treated mice determined by ELISA. (**C**) Representative photographs of the liver from each group (red circle indicated the obvious necrosis area). (**D**) Pathological analysis of liver sections. The top row showed the H&E staining images of liver sections (Scale bar = 200 μm). The middle row is the enlarged representative area from the upper image (Scale bar = 100 μm). The bottom row showed the pathology score of Con A-induced acidophilic degeneration, hepatocyte necrosis, and inflammatory cell infiltration in each group. Data are presented as the mean ± SD (*n* = 5). ## *p* < 0.01 and ### *p* < 0.001 were compared with the Control group. * *p* < 0.05, ** *p* < 0.01, and *** *p* < 0.001 were compared with the Con A-injected group.

A previous study has demonstrated that BSA-encapsulated AuNCs had extremely higher bioaccumulation in the liver than the GSH-protected AuNCs in normal mice [31]. Therefore, we compared the biodistribution and AIH therapeutic activity of HA and GA in Con A-induced AIH mice. The results indicated that the same dose of HA significantly increased liver accumulation in AIH mice compared to GA treatment (Figure 3A). A comparison of the therapeutic activity of HA and GA in AIH mice indicated that Con-A-induced elevations in plasma ALT and AST were greatly suppressed by GA or HA (Figure 3B). However, HA exhibited a higher inhibitory effect than GA, although the differences did not reach statistical significance (Figure 3B). Phenotype observation showed some obvious extensive flaky liver necrosis at the edges of the liver in mice stimulated by Con A, while no such phenomenon was observed in HA or GA treatment groups (Figure 3C). Multiple injections of Con A caused a significant increase in liver coefficient, which was alleviated by the HA or GA treatments, and HA showed more obvious regulation (Figure S3). The degree of liver damage was assessed in pathological sections of liver tissue, which were graded from 0 to 4 based on the presence of hepatocyte necrosis, eosinophilic degeneration, and inflammatory cell infiltration. A higher score indicates a more severe lesion. Results indicated that GA and HA treatment could significantly reduce hepatocyte necrosis, inflammatory infiltration, and hepatocyte degeneration caused by Con A-stimulation, and HA treatment showed a better effect (Figure 3D). These results suggested that HA enhanced the alleviating effect of AuNCs on Con A-induced AIH by improving liver targeting. 

### 3.4. HA Suppresses Con A-Induced Pro-Inflammatory Cytokines In Vivo

**Figure 4:**
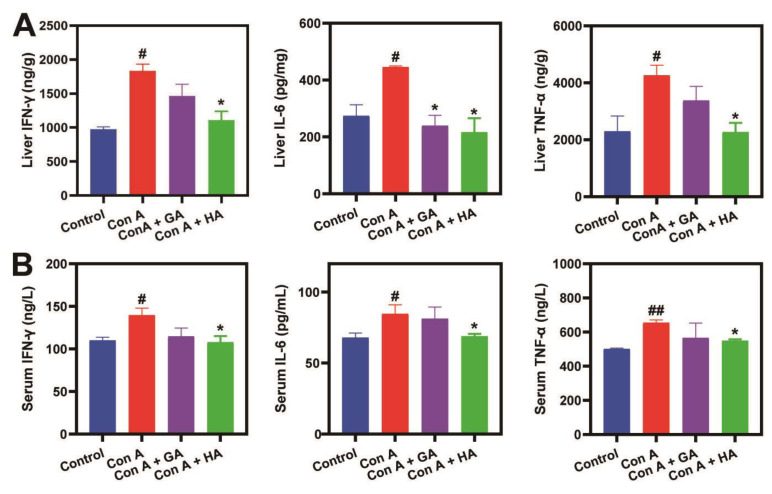
Regulation of cytokines by AuNCs in liver and serum of Con A-stimulated mice in vivo. (**A**) Levels of IFN-γ, TNF-α, and IL-6 in liver quantified by ELISA. (**B**) Levels of IFN-γ, TNF-α, and IL-6 in serum determined by ELISA. Data are presented as the mean ± SD (*n* = 5). # *p* < 0.05 and ## *p* < 0.01 were compared with the Control group. * *p* < 0.05 was compared with the Con A group.

The pro-inflammatory cytokines IFN-γ, TNF-α, and IL-6 produced by CD4^+^ T cells were identified to be the main culprits in Con A-mediated liver inflammation and damage [8]. To elucidate the underlying mechanisms of AuNCs in alleviating Con A-induced AIH, we examined the levels of IFN-γ, TNF-α, and IL-6 in the liver and serum of treated mice. As shown in Figure 4, GA or HA treatment reduced the elevated levels of IFN-γ, TNF-α, and IL-6 induced by Con A in the liver tissue, and HA exhibited more pronounced activity than GA (Figure 4A). In the serum, HA treatment also significantly suppressed the Con A-induced elevation of IFN-γ, TNF-α, and IL-6, while GA had no significant effect (Figure 4B). These results suggested that HA may enhance the suppression of Con A-induced pro-inflammatory cytokines by accumulating in the liver, thereby improving the remission of AIH.

### 3.5. Mechanism of HA Alleviating Con A-Induced AIH In Vitro

**Figure 5:**
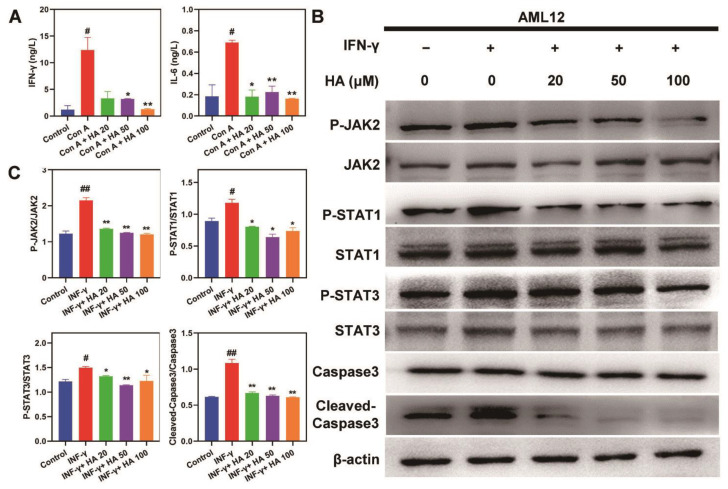
Mechanisms of HA alleviating Con A-induced AIH in vitro. Effects of different doses of HA (20, 50, 100 μM) on IFN-γ and IL-6. (**A**) Secretion in Con A-stimulated Jurkat cells determined by ELISA. Data are presented as the mean ± SD (*n* = 3). # *p* < 0.05 was compared with the Control group. * *p* < 0.05 and ** *p* < 0.01 were compared with the Con A-stimulated group. (**B**) Effects of HA on IFN-γ-induced activation of JAK2/STAT1 and STAT3, as well as the activated Caspase 3 in AML12 cells. The representative results of three repeated experiments. (**C**) The relative activation of JAK2, STAT1, STAT3, and Caspase3 in IFN-γ stimulated AML12 cells quantified by bands analysis in WB detections. Data are presented as the mean ± SD (*n* = 3). # *p* < 0.05 and ## *p* < 0.01 was compared with the Control group. * *p* < 0.05 and ** *p* < 0.01 were compared with the Con A-stimulated group.

After Con A is injected into mice, it directly causes vigorous CD4^+^ T-cell stimulation, leading to the expression and secretion of pro-inflammatory cytokines, including IFN-γ and IL-6 [36]. Then inflammatory macrophage infiltration is induced, and JAK/STAT (STAT1/STAT3) pathways in hepatocytes are activated, leading to apoptosis and immune-mediated liver injury [8]. Based on the results of cytokines analysis in serum and liver tissue, the molecular mechanisms of HA-mediated protection from Con A-induced AIH were explored in Jurkat and AML12 cell lines (T-cell and normal hepatocyte models, respectively) in vitro. Firstly, the cytotoxicity of HA to Jurkat and AML12 cells was assessed, and the results showed that HA did not affect the viability of these two cell lines even at a high dose of 100 μM Au (Figure S4). As shown in Figure 5A, HA treatment significantly inhibited the secretion of IFN-γ and IL-6 induced by Con A in Jurkat cells, even at a dose as low as 20 μM Au (Figure 5A). IFN-γ is the main cytokine responsible for STAT1 activation in hepatocytes [37]. IFN-γ/JAK/STAT plays an essential role in Con A-induced AIH via activating apoptotic signaling pathways [14]. As shown in Figure 5B,C, in IFN-γ-stimulated AML12 cells, HA can dose-dependently suppress JAK2-mediated activation of STAT1 and STAT3 pathways, as well as activation of the apoptotic executive protein Caspase 3, thereby protecting hepatocytes from apoptosis. These results demonstrated that HA could protect hepatocytes from Con A-induced apoptosis by inhibiting IFN-γ production in T cells and subsequent activation of the IFN-γ/STAT axis in hepatocytes.

### 3.6. HA Inhibits Con A-Induced Activation of STAT1/STAT3 In Vivo

**Figure 6:**
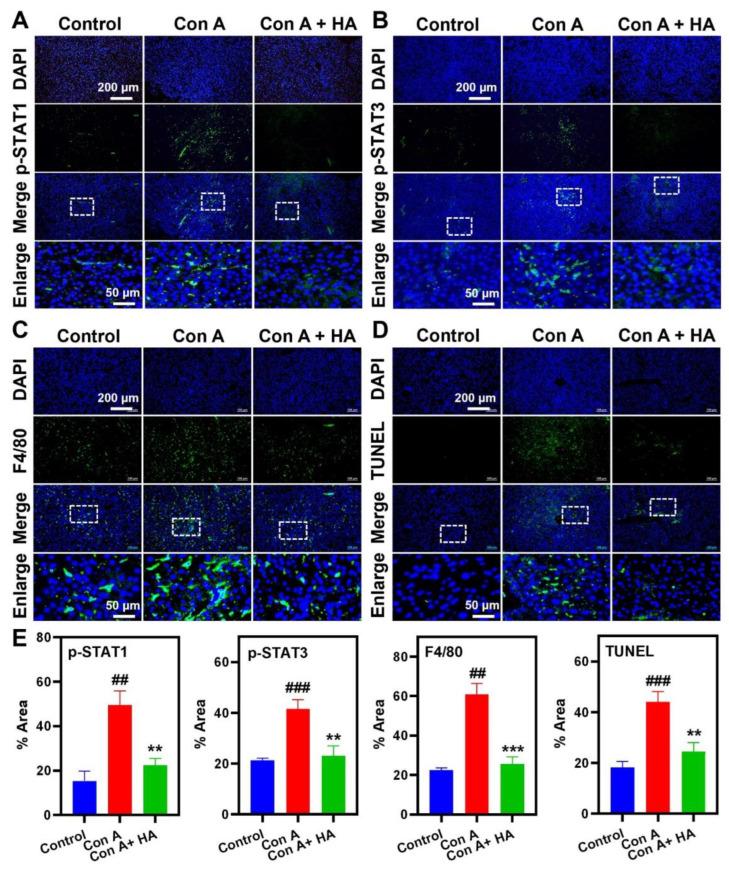
Immunofluorescence analysis of the mechanism of HA in liver sections after HA treatment. p-STAT1 (**A**), p-STAT3 (**B**), F4/80 (**C**), and TUNEL (**D**) were labeled in green fluorescence; the nucleus was stained in blue by DAPI. Representative confocal images are presented (scale bar = 200 μm). The bottom row of each panel is an enlargement of the area in the dashed box of the previous row (Scale bar = 50 μm). (**E**) Fluorescence intensity analysis of immunofluorescence staining results from (**A**–**D**). Data are presented as the mean ± SD (*n* = 5). ## *p* < 0.01 and ### *p* < 0.001 were compared with the Control group. ** *p* < 0.01 and *** *p* < 0.001 were compared with the Con A group.

According to the mechanism elucidated in cell lines, we further validated the effects of HA on Con A-induced activation of the STAT1/STAT3 pathways and macrophage infiltration in liver sections of Con A-injected mice by immunofluorescence staining. The consequential hepatocyte apoptosis was also detected by the terminal deoxynucleotidyl transferase dUTP nick-end labeling (TUNEL) assay. As shown in Figure 6, HA treatment significantly reduced the phosphorylation of STAT1 and STAT3 in the liver tissue induced by Con A-stimulation and effectively suppressed the infiltration of F4/80+ macrophages (Figure 6A–C). The TUNEL staining demonstrated that Con A-induced hepatocyte apoptosis was also markedly reduced by HA treatment, thereby protecting the liver tissue from immune-mediated damage caused by Con A (Figure 6D). Statistical analysis of fluorescence intensity confirmed the significance of these regulations (Figure 6E).

### 3.7. Biosafety of HA in Murine

**Figure 7:**
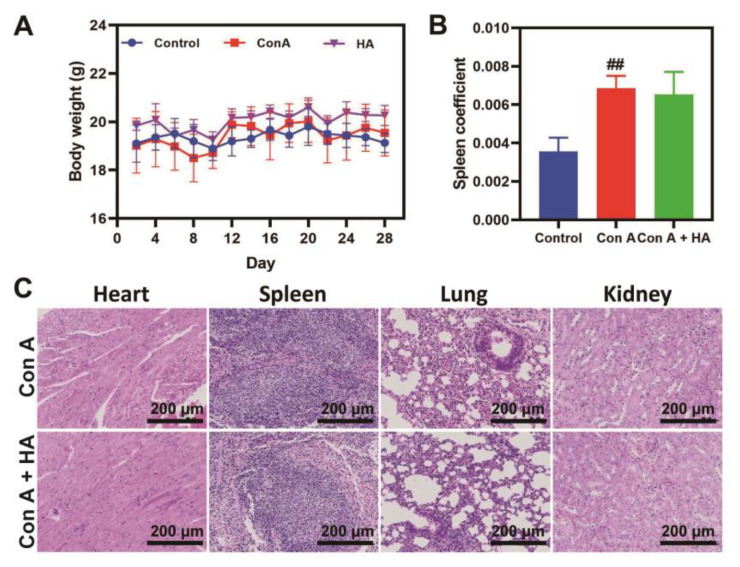
Biosafety evaluation of HA in treated mice. (**A**) Body weight monitoring of mice during 28 days of treatment. (**B**) Spleen coefficient after HA treatment. Data are presented as the mean ± SD (*n* = 5). ## *p* < 0.01 was compared with the Control group. (**C**) H&E staining of main organs (heart, spleen, lung, kidney) after HA treatment. (Scale bar = 200 µm).

Biodistribution analysis has shown that HA is mainly distributed in the spleen in addition to its accumulation in the liver; hence, the evaluation of its biosafety was crucial for its application (Figure 3A). Body weight monitoring showed that HA had no significant effect on weight gain in AIH mice over the course of 28 days of treatment (Figure 7A). The analysis of organ coefficients showed that HA treatment had no significant effect on the spleen coefficient of AIH mice, and the organ coefficients of the heart, lung, and kidney were also not significantly affected (Figure 7B and Figure S5). Further histological examination showed that HA administration did not cause significant organ damage, including the spleen, indicating its good biosafety (Figure 7C). The Pharmacokinetic of HA was further analyzed in rats. Results indicated that HA had a relatively long blood circulation time in vivo, and its elimination half-life (t_1/2z_) in female and male rats was 24.97 h and 38.84 h, respectively (Figure S6 and Table S1).

## 4. Conclusions

In summary, we employed GSH-protected AuNCs (GA) for the first time to unveil the potential therapeutic activity of bio-protected AuNCs in mitigating Con A-induced AIH in mice. On this basis, HSA-encapsulated AuNCs (HA), which exhibited enhanced liver targeting and AIH alleviation capabilities, were prepared. Furthermore, the underlying liver-protective mechanism of HA against Con A-induced liver injury was elucidated via the study of cell models and liver pathological sections. The results demonstrated that HA effectively suppressed inflammatory macrophage infiltration in the liver and hepatocyte apoptosis induced by Con A via inhibiting T-cell secretion of IFN-γ and IL-6, as well as subsequent IFN-γ-induced JAK/STAT pathway activation in hepatocytes, thereby alleviating immune-mediated liver injury. These findings highlight the promising potential of bio-protected AuNCs as a novel class of nanodrugs for AIH therapy, which can improve liver targeting via rational surface ligand engineering.

## Data Availability

Data will be made available on request.

## Supplementary Materials

The following supporting information can be downloaded at https://www.mdpi.com/article/10.3390/pharmaceutics16010110/s1, Figure S1: Characterization of GA; Figure S2: Zeta potential of HA; Figure S3: Liver coefficient of Con A-induced AIH mice after GA and HA treatment; Figure S4: Cytotoxicity of HA on (A) Jurkat cells and (B) AML12 cells after incubation for 24 h that evaluated by CCK-8; Figure S5: Organ coefficients of heart (A), lung (B) and kidney (C) after HA treatment; Figure S6: Concentrations of Au in the serum of male and female SD rat following intraperitoneal injection of HA at a dose of 7.5 mg Au·kg^−1^; Table S1: Values of parameters of the Pharmacokinetic of HA in rats.

## Abbreviations

AuNCs	Au nanoclusters
AIH	Autoimmune hepatitis
GSH	Glutathione
HSA	Human serum albumin
GA	GSH-protected AuNCs
HA	HSA-encapsulated AuNCs
Con A	Concanavalin A
DDS	Drug delivery system
AST	Aspartate transaminase
ALT	Alanine transaminase
INF-γ	Interferon gamma
STAT	Signal Transduction and Transcription Activator3
JAK2	Janus kinase2
TNF-α	Tumor necrosis factor
IL-6	Interleukin 6
FBS	Fetal bovine serum
DMEM	Dulbecco’s modified Eagle medium
CCK-8	Cell Counting Kit 8
PL	Photoluminescence
STEM	Scanning transmission electron microscope
EDS	Energy dispersive spectrum
ICP-MS	Inductively coupled plasma mass spectrometry
AML12	Alpha mouse liver 12
DMEM/F12	Dulbecco’s Modified Eagle Medium/Nutrient Mixture F-12
ECL	Enhanced chemiluminescence
ELISA	Enzyme-linked immunosorbent assay
H&E	Hematoxylin and Eosin
TUNEL	TdT-mediated dUTP Nick-End Labeling
PBS	Phosphate-buffered saline
SD	Standard deviation
DLS	Dynamic light dispersion
UV-VIS	Ultraviolet-visible
MALDI-TOF	Matrix-assisted laser desorption/Ionization time-of-flight

## References

[1] Muratori L., Lohse A.W., Lenzi M. (2023). Diagnosis and Management of Autoimmune Hepatitis. BMJ.

[2] Terziroli Beretta-Piccoli B., Mieli-Vergani G., Vergani D. (2017). Autoimmune Hepatitis: Standard Treatment and Systematic Review of Alternative Treatments. World J. Gastroenterol..

[3] Mieli-Vergani G., Vergani D., Czaja A.J., Manns M.P., Krawitt E.L., Vierling J.M., Lohse A.W., Montano-Loza A.J. (2018). Autoimmune Hepatitis. Nat. Rev. Dis. Primers.

[4] Pape S., Schramm C., Gevers T.J. (2019). Clinical Management of Autoimmune Hepatitis. United Eur. Gastroenterol. J..

[5] Brahim I., Brahim I., Hazime R., Admou B. (2017). Autoimmune Hepatitis: Immunological Diagnosis. Presse Med..

[6] Tiegs G., Hentschel J., Wendel A. (1992). A T Cell-Dependent Experimental Liver Injury in Mice Inducible by Concanavalin A. J. Clin. Investig..

[7] Sucher E., Sucher R., Gradistanac T., Brandacher G., Schneeberger S., Berg T. (2019). Autoimmune Hepatitis—Immunologically Triggered Liver Pathogenesis—Diagnostic and Therapeutic Strategies. J. Immunol. Res..

[8] Hao J., Sun W., Xu H. (2022). Pathogenesis of Concanavalin a Induced Autoimmune Hepatitis in Mice. Int. Immunopharmacol..

[9] Wang H.-X., Liu M., Weng S.-Y., Li J.-J., Xie C., He H.-L., Guan W., Yuan Y.-S., Gao J. (2012). Immune Mechanisms of Concanavalin a Model of Autoimmune Hepatitis. World J. Gastroenterol..

[10] Sass G., Heinlein S., Agli A., Bang R., Schümann J., Tiegs G. (2002). Cytokine Expression in Three Mouse Models of Experimental Hepatitis. Cytokine.

[11] Świerczek A., Plutecka H., Ślusarczyk M., Chłoń-Rzepa G., Wyska E. (2021). Pk/Pd Modeling of the Pde7 Inhibitor—Grms-55 in a Mouse Model of Autoimmune Hepatitis. Pharmaceutics.

[12] Cheng P., Chen K., Xia Y., Dai W., Wang F., Shen M., Wang C., Yang J., Zhu R., Zhang H. (2014). Hydrogen Sulfide, a Potential Novel Drug, Attenuates Concanavalin a-Induced Hepatitis. Drug Des. Dev. Ther..

[13] Kusters S., Gantner F., Kunstle G., Tiegs G. (1996). Interferon Gamma Plays a Critical Role in T Cell-Dependent Liver Injury in Mice Initiated by Concanavalin A. Gastroenterology.

[14] Tagawa Y.-I., Sekikawa K., Iwakura Y. (1997). Suppression of Concanavalin a-Induced Hepatitis in Ifn-Gamma (-/-) Mice, but Not in Tnf-Alpha (-/-) Mice: Role for Ifn-Gamma in Activating Apoptosis of Hepatocytes. J. Immunol..

[15] Li S., Xia Y., Chen K., Li J., Liu T., Wang F., Lu J., Zhou Y., Guo C. (2016). Epigallocatechin-3-Gallate Attenuates Apoptosis and Autophagy in Concanavalin a-Induced Hepatitis by Inhibiting Bnip3. Drug Des. Dev. Ther..

[16] Fan D., Cao Y., Cao M., Wang Y., Cao Y., Gong T. (2023). Nanomedicine in Cancer Therapy. Signal Transduct. Target. Ther..

[17] Yang G., Wang Z., Du F., Jiang F., Yuan X., Ying J.Y. (2023). Ultrasmall Coinage Metal Nanoclusters as Promising Theranostic Probes for Biomedical Applications. J. Am. Chem. Soc..

[18] Hao D., Zhang X., Su R., Wang Y., Qi W. (2023). Biomolecule-Protected Gold Nanoclusters: Synthesis and Biomedical Applications. J. Mater. Chem. B.

[19] Zare I., Chevrier D.M., Cifuentes-Rius A., Moradi N., Xianyu Y., Ghosh S., Trapiella-Alfonso L., Tian Y., Shourangiz-Haghighi A., Mukherjee S. (2021). Protein-Protected Metal Nanoclusters as Diagnostic and Therapeutic Platforms for Biomedical Applications. Mater. Today.

[20] Yuan Q., Wang Y., Zhao L., Liu R., Gao F., Gao L., Gao X. (2016). Peptide Protected Gold Clusters: Chemical Synthesis and Biomedical Applications. Nanoscale.

[21] Gao F., Yuan Q., Cai P., Gao L., Zhao L., Liu M., Yao Y., Chai Z., Gao X. (2019). Au Clusters Treat Rheumatoid Arthritis with Uniquely Reversing Cartilage/Bone Destruction. Adv. Sci..

[22] Lu C., Xue L., Luo K., Liu Y., Lai J., Yao X., Xue Y., Huo W., Meng C., Xia D. (2023). Colon-Accumulated Gold Nanoclusters Alleviate Intestinal Inflammation and Prevent Secondary Colorectal Carcinogenesis Via Nrf2-Dependent Macrophage Reprogramming. ACS Nano.

[23] Liu Y., Meng C., Li Y., Xia D., Lu C., Lai J., Zhang Y., Cao K., Gao X., Yuan Q. (2023). Peptide-Protected Gold Nanoclusters Efficiently Ameliorate Acute Contact Dermatitis and Psoriasis Via Repressing the Tnf-A/Nf-Κb/Il-17a Axis in Keratinocytes. Nanomaterials.

[24] Xia F., Hou W., Liu Y., Wang W., Han Y., Yang M., Zhi X., Li C., Qi D., Li T. (2018). Cytokine Induced Killer Cells-Assisted Delivery of Chlorin E6 Mediated Self-Assembled Gold Nanoclusters to Tumors for Imaging and Immuno-Photodynamic Therapy. Biomaterials.

[25] Feng D., Mei Y., Wang Y., Zhang B., Wang C., Xu L. (2008). Tetrandrine Protects Mice from Concanavalin a-Induced Hepatitis through Inhibiting Nf-Κb Activation. Immunol. Lett..

[26] Gu J., Li K., Lin H., Wang Y., Zhou Y., Chen D., Gu X., Shi H. (2023). Cadmium Induced Immunosuppression through Tlr-Iκbα-Nfκb Signaling by Promoting Autophagic Degradation. Ecotoxicol. Environ. Saf..

[27] Ren M., Zhang J., Dai S., Wang C., Chen Z., Zhang S., Xu J., Qin X., Liu F. (2023). Cx3cr1 Deficiency Exacerbates Immune-Mediated Hepatitis by Increasing Nf-Κb-Mediated Cytokine Production in Macrophage and T Cell. Exp. Biol. Med..

[28] Porret E., Le Guével X., Coll J.-L. (2020). Gold Nanoclusters for Biomedical Applications: Toward in Vivo Studies. J. Mater. Chem. B.

[29] Wong O.A., Hansen R.J., Ni T.W., Heinecke C.L., Compel W.S., Gustafson D.L., Ackerson C.J. (2013). Structure–Activity Relationships for Biodistribution, Pharmacokinetics, and Excretion of Atomically Precise Nanoclusters in a Murine Model. Nanoscale.

[30] Zhang B., Chen J., Cao Y., Chai O.J.H., Xie J. (2021). Ligand Design in Ligand-Protected Gold Nanoclusters. Small.

[31] Zhang X.-D., Wu D., Shen X., Liu P.-X., Fan F.-Y., Fan S.-J. (2012). In Vivo Renal Clearance, Biodistribution, Toxicity of Gold Nanoclusters. Biomaterials.

[32] Yasuda K., Maeda H., Kinoshita R., Minayoshi Y., Mizuta Y., Nakamura Y., Imoto S., Nishi K., Yamasaki K., Sakuragi M. (2023). Encapsulation of an Antioxidant in Redox-Sensitive Self-Assembled Albumin Nanoparticles for the Treatment of Hepatitis. ACS Nano.

[33] Mangano K., Cavalli E., Mammana S., Basile M.S., Caltabiano R., Pesce A., Puleo S., Atanasov A.G., Magro G., Nicoletti F. (2018). Involvement of the Nrf2/Ho-1/Co Axis and Therapeutic Intervention with the Co-Releasing Molecule Corm-A1, in a Murine Model of Autoimmune Hepatitis. J. Cell. Physiol..

[34] Xie J., Zheng Y., Ying J.Y. (2009). Protein-Directed Synthesis of Highly Fluorescent Gold Nanoclusters. J. Am. Chem. Soc..

[35] Zhang Z.C., Yao Y.W., Yuan Q., Lu C., Zhang X.C., Yuan J.L., Hou K.X., Zhang C.Y., Du Z.Y., Gao X.Y. (2020). Gold Clusters Prevent Breast Cancer Bone Metastasis by Suppressing Tumor-Induced Osteoclastogenesis. Theranostics.

[36] Crowley D., O’Callaghan Y., McCarthy A., Connolly A., Piggott C.O., FitzGerald R.J., O’Brien N.M. (2015). Immunomodulatory Potential of a Brewers’ Spent Grain Protein Hydrolysate Incorporated into Low-Fat Milk Following in Vitro gastrointestinal Digestion. Int. J. Food Sci. Nutr..

[37] Sang X.-X., Wang R.-L., Zhang C.-E., Liu S.-J., Shen H.-H., Guo Y.-M., Zhang Y.-M., Niu M., Wang J.-B., Bai Z.-F. (2017). Sophocarpine Protects Mice from Cona-Induced Hepatitis Via Inhibition of the Ifn-Gamma/Stat1 Pathway. Front. Pharmacol..

