# Supplementary material for: Enhancing Liver Delivery of Gold Nanoclusters via Human Serum Albumin Encapsulation for Autoimmune Hepatitis Alleviation
Source: Pharmaceutics. 2024 Jan 14;16(1):110. doi: 10.3390/pharmaceutics16010110 (PMC10818704; doi:10.3390/pharmaceutics16010110)
Supplement: Supplementary file 1 [file pharmaceutics-16-00110-s001.zip › pharmaceutics-2767899-supplementary.pdf]

pharmaceutics

MDPI

Supplementary Materials: Enhancing Liver Delivery of Gold Nanoclusters via Human Serum Albumin Encapsulation for Autoimmune Hepatitis Alleviation

Cong Meng, Yu Liu, Yuping Ming, Cao Lu, Yanggege Li, Yulu Zhang, Dongdong Su, Xueyun Gao and Qing Yuan

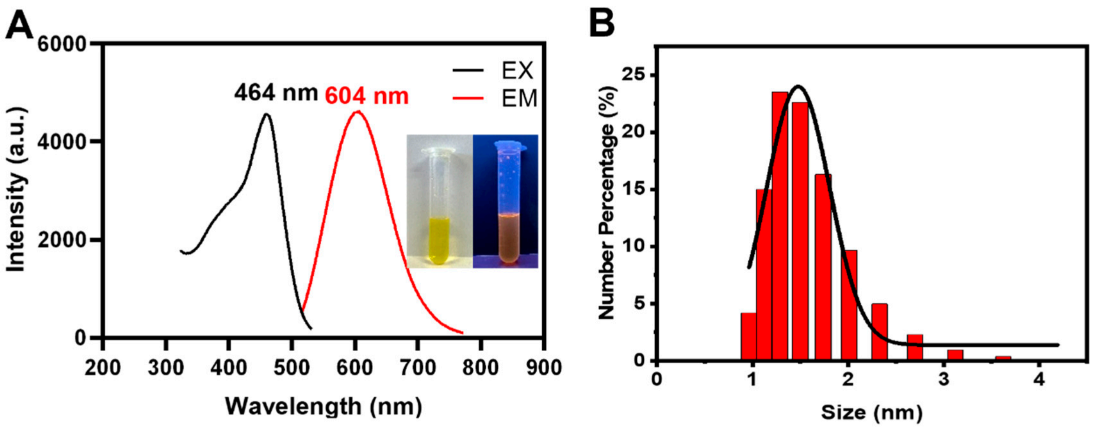

Figure S1. Characterization of GA. (A) The fluorescence spectra of GA, excitation peak at 464 nm and emission peak at 604 nm. Inset are the photos of GA solution under visible (left) and UV light (right). (B) Dynamic light scattering analysis of GA.

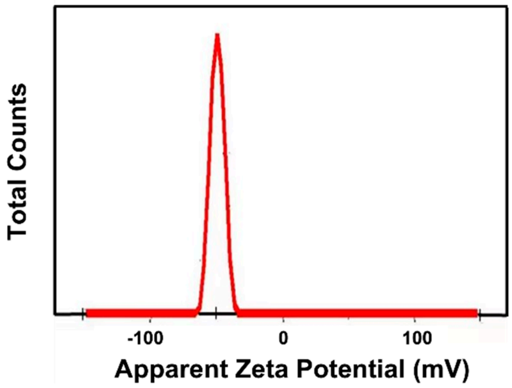

Figure S2. Zeta potential of HA.

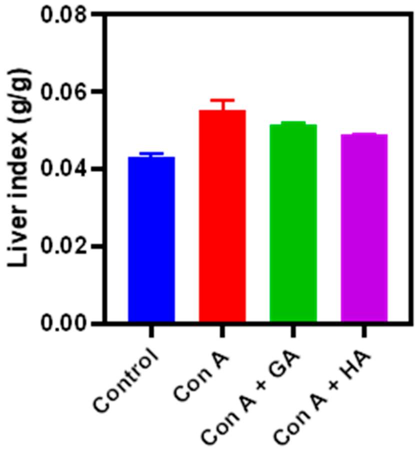

Figure S3. Liver index of Con A-induced AIH mice after GA and HA treatment.

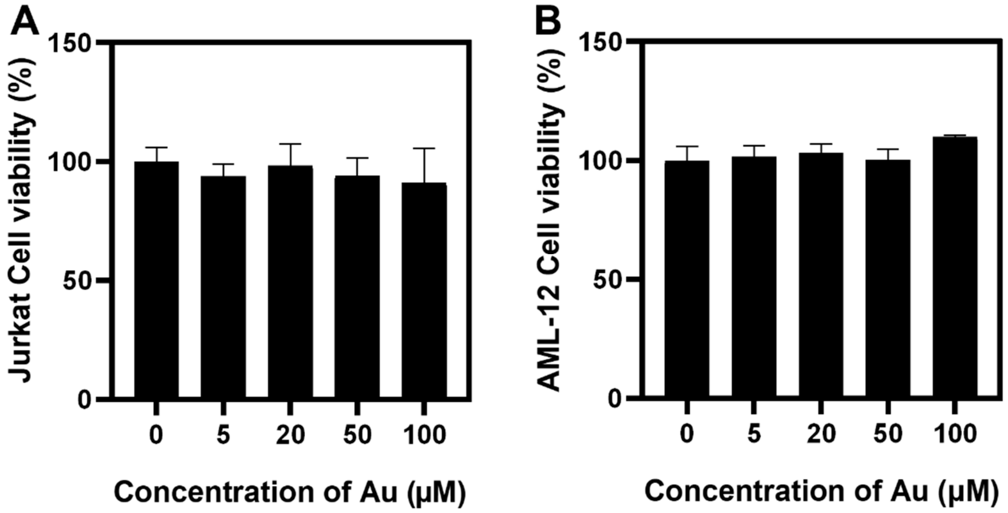

Figure S4. Cytotoxicity of HA on (A) Jurkat cells and (B) AML12 cells after incubation for 24 h that evaluated by CCK-8.

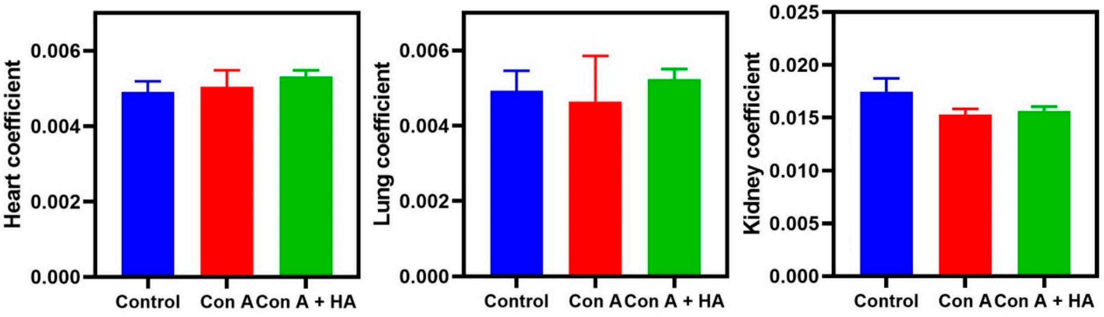

Figure S5. Organ coefficients of heart (A), lung (B) and kidney (C) after HA treatment.

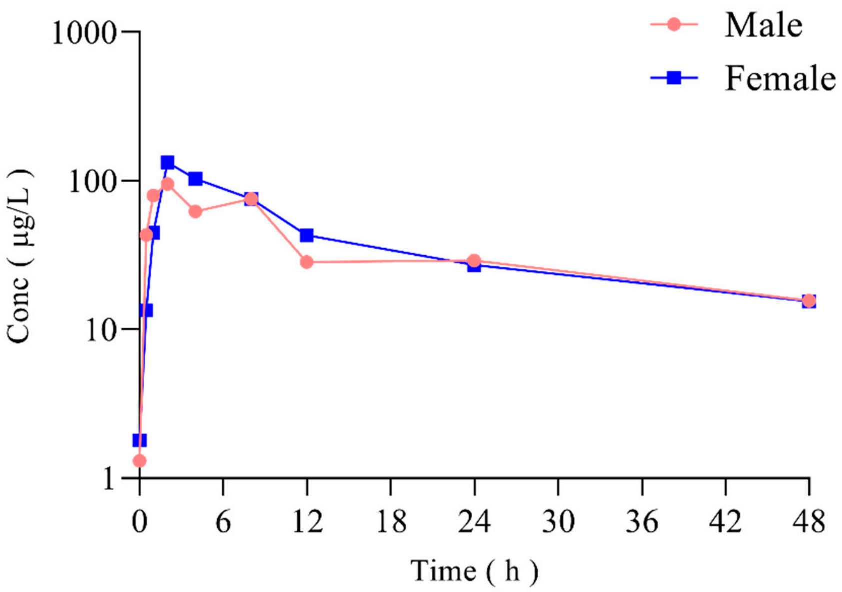

Figure S6. Concentrations of Au in the serum of male and female SD rat following intraperitoneal injection of HA at a dose of 7.5 mg Au·kg-1. Observed concentrations are shown as symbols, and the concentrations predicted by the model are shown as lines.

Table S1. Values of parameters of the Pharmacokinetic of HA in rats.

Parameter	Unit	Value	
		**Female**	**Male**
AUC(0-t)	μg/L*h	1839.28	1619.12
AUC(0-∞)	μg/L*h	2394.84	2496.70
AμMC(0-t)	h*h*ug/L	29853.10	28546.72
AμMC(0-∞)	h*h*ug/L	76535.90	119849.23
MRT(0-t)	h	16.23	17.63
MRT(0-∞)	h	31.96	48.00
t1/2z	h	24.97	38.84
Tmax	h	2	2
CLz/F	L/h/kg	3.13	3.00
Vz/F	L/kg	112.83	168.34
Cmax	μg/L	133.20	95.20
